# Genetic Polymorphisms and Sepsis in Premature Neonates

**DOI:** 10.1371/journal.pone.0101248

**Published:** 2014-07-07

**Authors:** Susanna Esposito, Alberto Zampiero, Lorenza Pugni, Silvia Tabano, Claudio Pelucchi, Beatrice Ghirardi, Leonardo Terranova, Monica Miozzo, Fabio Mosca, Nicola Principi

**Affiliations:** 1 Pediatric Highly Intensive Care Unit, Department of Pathophysiology and Transplantation, Università degli Studi di Milano, Fondazione IRCCS Ca' Granda Ospedale Maggiore Policlinico, Milan, Italy; 2 Neonatal Intensive Care Unit, Department of Clinical Sciences and Community Health, Università degli Studi di Milano, Fondazione IRCCS Ca' Granda Ospedale Maggiore Policlinico, Milan, Italy; 3 Genetic Unit, Department of Pathophysiology and Transplantation, Università degli Studi di Milano, Fondazione IRCCS Ca' Granda Ospedale Maggiore Policlinico, Milan, Italy; 4 Department of Epidemiology Unit, IRCCS Mario Negri Institute for Pharmacological Research, Milan, Italy; Charité-University Medicine Berlin, Germany

## Abstract

Identifying single nucleotide polymorphisms (SNPs) in the genes involved in sepsis may help to clarify the pathophysiology of neonatal sepsis. The aim of this study was to evaluate the relationships between sepsis in pre-term neonates and genes potentially involved in the response to invasion by infectious agents. The study involved 101 pre-term neonates born between June 2008 and May 2012 with a diagnosis of microbiologically confirmed sepsis, 98 pre-term neonates with clinical sepsis and 100 randomly selected, otherwise healthy pre-term neonates born during the study period. During the study, 47 SNPs in 18 candidate genes were genotyped on Guthrie cards using an ABI PRISM 7900 HT Fast real-time and MAssARRAY for nucleic acids instruments. Genotypes CT and TT of rs1143643 (the *IL1β* gene) and genotype GG of rs2664349GG (the *MMP-16* gene) were associated with a significantly increased overall risk of developing sepsis (p = 0.03, p = 0.05 and p = 0.03), whereas genotypes AG of rs4358188 (the *BPI* gene) and CT of rs1799946 (the *DEFβ1* gene) were associated with a significantly reduced risk of developing sepsis (p = 0.05 for both). Among the patients with bacteriologically confirmed sepsis, only genotype GG of rs2664349 (the *MMP-16* gene) showed a significant association with an increased risk (p = 0.02). Genotypes GG of rs2569190 (the *CD14* gene) and AT of rs4073 (the *IL8* gene) were associated with a significantly increased risk of developing severe sepsis (p = 0.05 and p = 0.01). Genotype AG of rs1800629 (the *LTA* gene) and genotypes CC and CT of rs1341023 (the *BPI* gene) were associated with a significantly increased risk of developing Gram-negative sepsis (p = 0.04, p = 0.04 and p = 0.03). These results show that genetic variability seems to play a role in sepsis in pre-term neonates by influencing susceptibility to and the severity of the disease, as well as the risk of having disease due to specific pathogens.

## Introduction

Despite significant advances in supportive care, neonatal sepsis continues to be a major cause of morbidity and mortality, particularly among premature infants. It occurs in 1/1,000 full-term and 4/1,000 premature live births, and mortality rates can reach values up to 20% in some settings and among very low-birth-weight (VLBW) infants [1]–[3].

Susceptibility to, and the severity and outcome of sepsis depend on various factors, including environmental exposure, host immune status and inflammatory responses. Over the last few years, it has been shown that these interacting factors can be modified by variations in gene function or expression that can lead to unexpected individual responses to infection [4]–[6]. Most of the research in this regard has concentrated on the potential association between such responses and host genetic variability in the regulatory and coding region of genes for components of innate and adaptive immunity in adults and older children, but rarely infants [7].

There are therefore few data concerning the effects of genetic variations on the risk of developing, severity and outcome of early- and late-onset sepsis in neonates, although some reports suggest that they may be related [8]–[10].

However, a more rigorous evaluation of the possible association between genetic variations and neonatal sepsis is particularly important because of newborn infants have an immature immune system, and studies of their innate and adaptive responses have demonstrated that some aspects of innate immunity to bacterial infection are impaired, particularly in VLBW infants [11], [12]. This *per se* may predispose to more frequent and/or more severe sepsis. Identifying genetic variations in the genes involved in bacteria-induced cell responses and those involved in the pathogenesis of sepsis may help to clarify the pathophysiology of sepsis in this group of high-risk patients, and this could lead to the development of new diagnostic tools, improved specific therapeutic measures, and the more accurate prediction of patient outcomes.

The aim of this study was to evaluate the relationships between sepsis in pre-term neonates and 47 genetic variants in 18 genes potentially involved in the response to invasion by infectious agents.

## Methods

### Study design

This retrospective study involved pre-term infants (<37 weeks' gestation) admitted to the Neonatal Intensive Care Unit of the Fondazione IRCCS Ca' Granda Ospedale Maggiore Policlinico, Milan, Italy, between June 2008 and May 2012. The study was approved by the Ethics Committee of the Fondazione IRCCS Ca' Granda Ospedale Maggiore Policlinico. Moreover, two of us (LP and BG) informed parents or legal guardian of the study as well as obtained written informed consent for the use of clinical data and blood samples of each child who could be enrolled before the study was begun.

Three groups of pre-term infants were enrolled. The first group consisted of 101 pre-term neonates with culture-proven sepsis (i.e. with signs and symptoms of clinical sepsis associated with at least one blood culture that was positive for a bacterial pathogen) Blood cultures positive for following microorganisms generally considered to be contaminants, including *Corynebacterium* spp., *Propionibacterium* spp., and *Penicillium* spp., were excluded from analysis. The diagnosis of sepsis due to coagulase-negative *Staphylococcus* (CoNS) was based on the criteria of the Vermont Oxford Network Database [13] and required clinical signs of sepsis, two blood culture positive for CoNS and intravenous antibacterial therapy for at least 5 days after performing blood culture, or until death. Whenever CoNS and another pathogen were identified in the same blood culture, only the other pathogen was considered the pathogen. The second consisted of 100 pre-term neonates with signs and symptoms of clinical sepsis but negative blood culture(s) during the observation period. The neonates in both groups systematically received antibiotic therapy for ≥7 days on the basis of the findings of microbiological sensitivity tests (when available) or the recommendations of the international guidelines [14]. The third group consisted of 100 pre-term neonates who did not have any respiratory problems, never had a positive blood culture, and never received antibiotic therapy during hospitalisation. The neonates in each group were randomly selected on the basis of a computer-generated randomisation list from among those hospitalised in the Neonatal Intensive Care Unit during the study period. The exclusion criteria were premature infants with birth defects and those born of pregnancies leading to twins or higher multiples.

In accordance with the Report on the Expert Meeting on Neonatal and Paediatric Sepsis (8 June 2010, EMA, London) [15], clinical sepsis was defined as the presence of at least two clinical and two laboratory criteria in the previous 24 hours. The c*linical criteria* were 1) hyper- or hypothermia or temperature instability; 2) reduced urinary output or hypotension or mottled skin or impaired peripheral perfusion; 3) apnea or increased oxygen requirement or an increased requirement for ventilator support; 4) episodes of bradycardia or tachycardia or rhythm instability; 5) feeding intolerance or abdominal distension; 6) lethargy or hypotonia or irritability; and 7) skin and subcutaneous lesions such as petechial rash or sclerema. The *laboratory criteria* were: 1) a white blood cell (WBC) count of <4 or >20×10^9^ cells/L; 2) an immature to total neutrophil ratio (I/T) of >0.2; 3) a platelet count of <100×10^9^/L; 4) C-reactive protein (CRP) levels of >15 mg/L or procalcitonin levels of ≥2 ng/mL; 5) glucose intolerance when receiving normal amounts of glucose (8–15 g/kg/day) as expressed by blood glucose values of >180 mg/dL or hypoglycemia (<40 mg/dL) confirmed at least twice; and 6) acidosis as characterised by a base excess (BE) of <−10 mmol/L or lactate levels of >2 mmol/L.

The clinical, laboratory and outcome data were obtained from the Neonatal Intensive Care Database, whereas genetic evaluations were made using blood extracted from filter Guthrie cards prepared at birth as part of our routine clinical practice, not used for the screening of inborn errors of metabolism, and archived in an envelope.

In accordance with criteria of Goldstein *et al.* [16], sepsis was defined severe in the presence of shock, cardiovascular organ dysfunction or acute respiratory distress syndrome, or two or more other organ dysfunctions, or death.

### Candidate genes

A total of 47 SNPs of 18 candidate genes involved in immune regulation and the pathogenesis of inflammation and sepsis were selected for analysis (see Table 1). The genes encode pattern recognition receptors (CD14, TLR2, and TLR4), intracellular signalling proteins (IRAK1), pro-inflammatory cytokines (IL1α, IL1β IL6, and LTA), anti-inflammatory cytokines (IL10), chemokines (IL8, CXCL10), bactericidal-permeability increasing protein (BPI), mannose binding lectin-2 (MBL2), beta-defensin1 (DEFβ1), matrix metalloproteinase-16 (MMP-16), serpine1, heat shock protein12A (HSPA12A), and ring finger protein 175 (RNF175). All are located on autosomes except *IRAK1*, which is located on the X chromosome. Most of the SNPs are functional variants or tagging SNPs characterised by the International HapMap Project: some are known to be involved in the onset, severity or outcome of sepsis in experimental animals or humans [4]–[6], and the others have been previously found to be associated with an increased risk of developing specific infections or an abnormal immune response [17]–[20].

**Table 1:** Gene and single nucleotide polymorphisms (SNPs).

Gene	dbSNP	HGVS description	Functional consequence	Position (bp)	Chr	Gene location
*TLR2*	Rs11938228	NG_016229.1:g.21506C>A	Intron variant	154621946	4	Intron
	Rs4696480	NG_016229.1:g.6686T>A	Intron variant	154607126	4	Intron
	Rs5743708	NG_016229.1:g.25877G>A	Missense	154626317	4	Exon
	Rs3804099	NG_016229.1:g.24216T>C	Synonymous codon	154624656	4	Exon
	Rs3804100	NG_016229.1:g.24969T>C	Synonymous codon	154625409	4	Exon
*TLR4*	Rs1927911	NG_011475.1:g.8595A>G	Intron variant	120470054	9	Intron
	Rs2149356	NG_011475.1:g.12740T>G	Intron variant	120474199	9	Intron
	Rs4986790	NG_011475.1:g.13843A>G	Missense	120475302	9	Exon
	Rs4986791	NG_011475.1:g.14143C>T	Missense	120475602	9	Exon
	Rs1554973	NG_011475.1:g.19353T>C	Transition substitution	120480812	9	Intergenic
*CD14*	Rs2569190	NG_023178.1:g.5371T>C	Intron variant, UTR variant 5′	140012916	5	UTR 5′
*Ring Finger Protein 175*	Rs1585110	NG_016386.1:g.25444G>A	Intron variant	154660944	4	Intron
*IRAK1*	Rs1059703	NG_008387.1:g.11514C>T	Intron variant, missense	153278829	X	Intron
	Rs3027898	NG_008387.1:g.14453G>T	Downstream variant, intron variant	153275890	X	Intergenic
*IL1α*	Rs1800587	NG_008850.1:g.5012C>T	UTR variant 5′	113542960	2	UTR 5′
*IL1β*	Rs1143643	NG_008851.1:g.11055G>A	Intron variant	113588302	2	Intron
	Rs1143633	NG_008851.1:g.8890G>A	Intron variant	113590467	2	Intron
	Rs1143627	NG_008851.1:g.4970C>T	Upstream variant 2KB	113594387	2	Intron
	Rs16944	NG_008851.1:g.4490T>C	Upstream variant 2KB	113594867	2	Intron
*IL6*	Rs1800797	NG_011640.1:g.4456A>G	Upstream variant 2KB	22766221	7	Intron
	Rs1554606	NG_011640.1:g.6942T>G	Intron variant,upstream variant 2KB	22768707	7	Intron
*IL8*	Rs4073	NG_029889.1:g.4802A>T	Upstream variant 2KB	74606024	4	Intergenic
*IL10*	Rs1800872	NG_012088.1:g.4433A>C	Upstream variant 2KB	206946407	1	Intergenic
	Rs1800896	NG_012088.1:g.3943A>G	Upstream variant 2KB	206946897	1	Intergenic
	Rs1800871	NG_012088.1:g.4206T>C	Upstream variant 2KB	206946634	1	Intergenic
*CXCL-10*	Rs8878	NM_001565.3:c.*783T>C	Intron variant, UTR variant 3′	76942300	4	UTR 3′
	Rs3921	NM_001565.3:c.*140G>C	Intron variant, UTR variant 3′	76942943	4	UTR 3′
	Rs4859587	NM_001565.3:c.279-195T>G	Intron variant	76943296	4	Intron
	Rs4859588	NM_001565.3:c.189-69C>T	Intron variant	76943677	4	Intron
*LTA*	Rs1800629	NG_012010.1:g.8156G>A	Upstream variant 2KB	31543031	6	Intergenic
	Rs1799964	NG_012010.1:g.7433T>C	Downstream variant 500B	31542308	6	Intergenic
	Rs2229094	NG_012010.1:g.5681T>C	Missense	31540556	6	Exon
	Rs1041981	NG_012010.1:g.5909C>A	Missense	31540784	6	Exon
*MBL2*	Rs5030737	NG_008196.1:g.5219C>T	Missense	54531242	10	Exon
	Rs7096206	NG_008196.1:g.4776C>G	Upstream variant 2KB	54531685	10	Intron
	Rs1800451	NG_008196.1:g.5235G>A	Missense	54531226	10	Exon
	Rs1800450	NG_008196.1:g.5226G>A	Missense	54531235	10	Exon
*BPI*	Rs4358188	NM_001725.2:c.646G>A	Missense	36946848	20	Exon
	Rs1341023	NM_001725.2:c.47C>T	Missense	36932660	20	Exon
	Rs5743507	NM_001725.2:c.546G>C	Synonymous codon	36939052	20	Exon
	Rs2232578	NM_004139.3:c.-205A>G	Upstream variant 2KB	36974715	20	Intergenic
*Serpin- α1*	Rs7242	NG_013213.1:g.16067T>G	UTR variant 3′	100781445	7	UTR 3′
*DEF-β1*	Rs11362	NM_005218.3:c.-20G>A	UTR variant 5′	6735399	8	UTR 5′
	Rs1799946	NM_005218.3:c.-52G>A	UTR variant 5′	6735431	8	UTR 5′
	Rs2741136	NM_005218.3:c.-1817T>C	Upstream variant 2KB	6737196	8	Intergenic
*MMP-16*	Rs2664349	NM_005941.4:c.1084-2311C>T	Intron variant	89089282	8	Intron
*HSPA-12A*	Rs740598	NT_030059.13:g.69311363G>A	Intron variant	118506899	10	Intron

Bp = base pairs; chr: chromosome; HGVS: Human Genome Variation Society. The position reflects the distance from the short-arm telomere.

### DNA extraction and genotyping

The blood spots on filter paper were cut into 3 mm punches using a Harris UniCore punch (Whatman, Milan, Italy), and stored in Eppendorf polypropylene tubes until use. Two punches were used for the extraction with Masterpure DNA Purification kit (Epicentre, Madison, FL, USA) according to the manufacturer's instructions and using 50 mcL final elution volume after purification. The DNA extracted was quantified using Picogreen reagent (Life Technologies, Monza, Italy) and an Infinite M200 PRO fluorimeter (Tecan Italia, Cernusco sul Naviglio, Italy). Following nucleic acid purification procedures, samples were stored at −20°C until use.

The SNPs were genotyped using the Custom TaqMan Array Microfluidic Cards genotyping system on an ABI 7900HT (Applied Biosystems, Foster City, CA). After PCR amplification, the alleles were detected by means of end-point analysis using SDS software and TaqMan Genotyper software (Applied Biosystems). The genotype data were entered into a Progeny database (Progeny Software, LLC, South Bend, IN) for the generation of datasets for analysis. However, because the Taqman genotyping approach failed in the identification of 11 of the 47 selected SNPs (rs4859588, rs1800896, rs2569190, rs3921, rs1800871, rs4986790, rs4859587, rs1800872, rs1143633, rs1800587, rs8878, respectively) mass spectrometry was used to complete the study.

### Mass spectrometry

The PCR and extension primers were designed using the Assay Design suite, version 1.0 (Sequenom, Inc., San Diego, CA, USA), and simultaneously detected 11 SNPs in a multiplex amplification reaction. Between 10 and 30 ng of genomic DNA were amplified by PCR by means of 45 2-minute cycles (95°C for 30 s, 56°C for 30 s, and 72°C for 60 s), followed by 72°C for 5 min, and finally 4°C. The final concentration of each PCR primer was 0.1 mcM and the final reaction volume was 5 mcL. Subsequently, the excess dNTPs of the PCR products were removed by means of treatment with 0.5 U shrimp alkaline phosphatase at 37°C for 40 min and 85°C for 5 min. Single-base extensions were performed in accordance with the manufacturer's instructions: 94°C for 30 s [94°C for 5 s, (52°C for 5 s, 80°C for 5 s) for 5 cycles] for 40 cycles, 72°C for 3 min, and then 4°C. After desalting, the reaction products were spotted for detection in a mass spectrometer (Sequenom's MassARRAY), and the data were analysed using Typer version 4.0 software (Sequenom).

### Statistical analysis

Genotype frequencies were calculated by means of direct counting. In order to investigate Hardy-Weinberg equilibrium (HWE), we compared the expected and observed numbers of different genotypes, and assessed potential deviations using the chi-squared test or likelihood ratio as appropriate. Univariate odds ratios (OR) and their 95% confidence intervals (CI) were calculated in order to measure the associations between selected SNPs and: 1) susceptibility to sepsis by comparing all children with sepsis (regardless of bacteriological confirmation) and controls; 2) susceptibility to bacteriologically confirmed sepsis; 3) susceptibility to severe sepsis; and 4) susceptibility to Gram-positive sepsis. The data were controlled for multiple testing using the false discovery rate method (with the Benjamini-Hochberg procedure). All of the statistical analyses were made using SAS software, version 9.2 (Cary, NC, USA).

## Results

During the study period, the parents of two premature neonates in the group with clinical sepsis and a negative blood culture withdrew their authorisation to use their children's blood and clinical data. Consequently, the results refer to 101 children with microbiologically confirmed sepsis, 98 patients with clinical sepsis and no positive blood culture, and 100 controls. Table 2 shows the demographic and clinical characteristics of the three groups, which were perfectly comparable in terms of gestational age, birth weight, gender, ethnicity and cesarean delivery. The neonates with microbiologically confirmed or clinical sepsis required mechanical ventilation significantly more frequently (p<0.05) and had a significantly worse outcome (p<0.05) than the controls, thus confirming the importance of sepsis in conditioning the final outcome. However, there was no difference in these variable between the two sepsis groups. The children with microbiological or clinical sepsis had late-onset sepsis (>72 hours) occurring at an average age of respectively 24 and 26 days.

**Table 2:** Demographic and clinical characteristics of the study groups.

Characteristic	Culture-proven sepsis (n = 101)	Clinical sepsis (n = 98)	Controls (n = 100)
Median gestational age, weeks (range)	28 (23–36)	28 (24–36)	30 (24–36)
Median birth weight, g (range)	1,040 (470–3,750)	1,000 (360–3,820)	1,310 (420–3,000)
Males (%)	52 (51.5)	53 (54.1)	50 (50.0)
Ethnicity, n (%)			
Caucasian	91 (90.1)	86 (87.8)	91 (91.0)
African	4 (4.0)	6 (6.1)	4 (4.0)
Asian	6 (5.9)	6 (6.1)	5 (5.0)
Cesarean delivery, n (%)	60 (59.4)	61 (62.2)	58 (58.0)
Ventilation required, n (%)	87 (86.1)*	71 (72.4)*	9 (9.0)
Negative outcome, n (%)	31 (30.7)*	22 (22.4)*	6 (6.0)
Severe sepsis	21	10	0
Death	10	12	6

*p<0.05 *vs* controls; no other significant between-group difference.

Table 3 lists the bacterial pathogens identified in the premature neonates with a positive blood culture. Gram-positive organisms (mainly CoNS) were cultured in 67.3% of cases, and Gram-negative rods (mainly *Escherichia coli*) were identified in the remaining 32.7%.

**Table 3:** Distribution of pathogens in the blood cultures of 101 neonates with microbiologically-confirmed sepsis.

Pathogen	No. (%)
Gram-positive infection	68 (67.3)
Coagulase-negative *Staphylococcus*	34
*Staphylococcus aureus*	16
*Enterococcus* spp.	12
*Streptococcus agalactiae*	6
Gram-negative infection	31 (30.7)
*Escherichia coli*	16
*Klebsiella* species	6
*Serratia* spp.	5
*Pseudomonas* spp.	4

All of the examined SNPs were present in the study population. Table 4 shows the SNPs with significantly different genotype frequencies between the neonates with bacteriologically confirmed or clinical sepsis and the controls, and Table 5 those that were significantly different between the neonates with bacteriologically confirmed sepsis and controls. Genotypes CT and TT of IL1β-rs1143643 and GG of MMP-16-rs2664349 were associated with a significantly increased overall risk of developing sepsis (p = 0.03, p = 0.05 and p = 0.03), whereas genotypes AG of BPI-rs4358188 and CT of DEFβ1-rs1799946 were associated with a significantly reduced risk (p = 0.05 for both). Only GG genotype of MMP-16-rs2664349 showed a significant association with an increased risk of developing bacteriologically confirmed sepsis (p = 0.02).

**Table 4:** Genotype frequencies with significant differences in the selected SNPs between controls and children with sepsis.^a^

Gene and polymorphic alleles	Control group (n = 100)		Children with sepsis (n = 199)		HWE, χ^2^ Controls	HWE, χ^2^ Sepsis	Outcome		
	N	%	N	%	p-value	p-value	OR	95% CI	p-value^b^
**IL-1 β-rs1143643**									
C	52	54.7	75	38.9			1	(reference)	
C/T	33	34.7	86	44.6			1.81	(1.06–3.09)	0.03
T	10	10.5	32	16.6	0.18	0.39	2.22	(1.00–4.90)	0.05
**BPI-rs4358188**									
A	20	20.2	40	20.1			0.70	(0.34–1.40)	0.31
A/G	54	54.6	87	43.7			0.56	(0.32–0.99)	0.05
G	25	25.3	72	36.2	0.35	0.15	1	(reference)	
**DEF- β1-rs1799946**									
C	28	29.2	79	40.3			1	(reference)	
C/T	49	51.0	78	39.8			0.56	(0.32–0.99)	0.05
T	19	19.8	39	19.9	0.77	0.02	0.73	(0.36–1.46)	0.37
**MMP-16-rs2664349**									
A	49	50.0	90	47.1			1	(reference)	
A/G	45	45.9	75	39.3			0.91	(0.55–1.51)	0.71
G	4	4.1	26	13.6	0.11	0.11	3.54	(1.17–10.72)	0.03

a The sums may not add up to the total because of some missing values. HWE: Hardy-Weinberg equilibrium.

b p-values from univariate analyses, not adjusted for multiple testing. None of the p-values was significant after correction for multiple testing.

**Table 5:** Genotype frequencies with significant differences in the selected SNPs between controls and children with bacteriologically confirmed (BC) sepsis.^a^

Gene and polymorphic alleles	Control group (n = 100)		Children with BC sepsis (n = 101)		HWE, χ^2^ Controls	HWE, χ^2^ BC sepsis	Outcome		
	N	%	N	%	p-value	p-value	OR	95% CI	p-value^b^
**DEF- β1-rs1799946**									
C	28	29.2	43	43.4			1	(reference)	
C/T	49	51.0	37	37.4			0.49	(0.26–0.93)	0.03
T	19	19.8	19	19.2	0.77	0.04	0.65	(0.29–1.44)	0.29
**MMP-16-rs2664349**									
A	49	50.0	43	44.3			1	(reference)	
A/G	45	45.9	40	41.2			1.01	(0.56–1.83)	0.97
G	4	4.1	14	14.4	0.11	0.35	3.99	(1.22–13.04)	0.02

a The sums may not add up to the total because of some missing values. HWE: Hardy-Weinberg equilibrium.

b p-values from univariate analyses, not adjusted for multiple testing. None of the p-values was significant after correction for multiple testing.

Table 6 shows the differences in SNP genotype frequencies between the neonates with severe and non-severe sepsis. GG genotype of CD14-rs2569190 and AT genotype of IL8-rs4073 were associated with a significantly increased risk of developing severe sepsis (p = 0.05 and p = 0.01).

**Table 6:** Genotype frequencies with significant differences in the selected SNPs between children with non-severe and those with severe sepsis.^a^

Gene and polymorphic alleles	Non-severe sepsis (n = 133)		Severe sepsis (n = 66)		HWE, χ^2^ Non-severe	HWE, χ^2^ Severe	Outcome		
	N	%	N	%	p-value	p-value	OR	95% CI	p-value^b^
**CD14-rs2569190**									
A	38	30.7	14	21.9			1	(reference)	
A/G	63	50.8	30	46.9			1.29	(0.61–2.74)	0.50
G	23	18.6	20	31.3	0.73	0.66	2.36	(1.00–5.56)	0.05
**IL8-rs4073**									
A	28	21.7	14	21.2			1.82	(0.76–4.36)	0.18
A/T	50	38.8	38	57.6			2.77	(1.34–5.72)	0.01
T	51	39.5	14	21.2	0.02	0.22	1	(reference)	

a The sums may not add up to the total because of some missing values. HWE: Hardy-Weinberg equilibrium.

b p-values from univariate analyses, not adjusted for multiple testing. None of the p-values was significant after correction for multiple testing.

Table 7 shows the differences in SNP genotype frequencies between the neonates with Gram-negative or Gram-positive sepsis. Genotypes AG of LTA-rs1800629 and CC and CT of BPI-rs1341023 were associated with a significantly increased risk of developing Gram-negative sepsis (p = 0.04, p = 0.04 and p = 0.03).

**Table 7:** Genotype frequencies with significant differences in the selected SNPs between children with Gram-negative and those with Gram-positive sepsis.^a^ ^,^ ^b^

Gene and polymorphic alleles	Gram− sepsis (n = 31)		Gram+ sepsis (n = 68)		HWE, χ^2^ Gram−	HWE, χ^2^ Gram+	Outcome		
	N	%	N	%	p-value	p-value	OR^c^	95% CI	p-value^d^
**LTA-rs1800629**									
A	1	3.2	0	0.0			<0.001	-	-
A/G	12	38.7	13	19.4			0.36	(0.14–0.93)	0.04
G	18	58.1	54	80.6	0.55	0.38	1	(reference)	
**BPI-rs1341023**									
C	7	23.3	9	13.2			0.25	(0.07–0.93)	0.04
C/T	17	56.7	28	41.2			0.32	(0.11–0.92)	0.03
T	6	20.0	31	45.6	0.46	0.51	1	(reference)	

a The sums may not add up to the total because of some missing values.

b Two subjects had fungal infections and were not included in this analysis.

c Odds ratios of Gram-positive sepsis. HWE: Hardy-Weinberg equilibrium.

d p-values from univariate analyses, not adjusted for multiple testing. None of the p-values was significant after correction for multiple testing.

There were no other differences in the studied allele and genotype frequencies between the neonates with sepsis (overall or bacteriologically confirmed) and controls, or between those with severe or non-severe sepsis, or between those with Gram-positive or Gram-negative sepsis.

## Discussion

Identifying genetic variants that can predict human susceptibility to, and outcomes of sepsis may help to identify patients at higher risk of death or serious complications who require prompt and aggressive therapy. This is extremely important in premature neonates, who are at highest risk of developing poorly controllable severe bacterial infections for a number of reasons. Susceptibility to sepsis in our study population was related to SNPs in the *IL1β*, *MMP-16*, *BPI*, and *DEFβ1* genes. However, whereas SNPs in the *IL1β* and in *MMP-16* genes were associated with an increased risk of sepsis, variations of *BPI* and *DEFβ1* seemed to play a protective role.

The potential role of a genetic alteration in the *IL1β* gene in favouring the development of sepsis in premature infants found in this study is in conflicts with the findings of Abu-Maziad *et al.* who did not find any association [8]. This discrepancy may be explained by differences in the definition of sepsis and its severity, and in the general characteristics of the enrolled subjects, including ethnicity. On the other hand, conflicting results concerning the influence of other IL1β SNPs on the development and evolution of various infectious diseases have been repeatedly reported [18], [21]–[24]. Most of the sepsis data have been collected in studies of rs16944, and Ma *et al.* [21] and Fang *et al.* [24] did not find any correlation between it and susceptibility to sepsis in adults, whereas Read *et al.* found that it was associated with increased survival of in a group of mainly pediatric patients with meningococcemia [22]. Taken together, these findings indicate that further studies are needed to clarify whether and which SNPs of a gene that codes for a factor, IL1β, which plays an important role in the pathogenesis of sepsis and septic shock, are really important in conditioning the development and outcome of the disease [25].

We found that homozygosis for rs2664349-GG haplotype in the *MMP-16* gene is associated with an increased susceptibility to sepsis in general and to microbiological confirmed sepsis in particular. This is the first report of the potential effect of a genetic variation in MMP-16 on sepsis, but the finding seems to be consistent with recent evidence that MMPs are not only purely matrix-degrading enzymes as previously thought, but also have multiple immunomodulation mechanisms [26]. Although the range of infectious diseases, the organs involved, and the nature of the resulting tissue damage vary depending on the type of MMP, all of them play a role in facilitating leukocyte recruitment, cytokine and chemokine processing, defensin activation, and matrix remodelling [27]. It has also been found that excess MMP activity following infection may lead to an immunopathology that causes host morbidity or mortality and favours pathogen dissemination or persistence [26]. The possibility that MMP genetic variations can significantly influence susceptibility to, and the course and outcome of infectious diseases in humans has been little studies so far. In the case of sepsis, Chen *et al.* studied seven frequent SNPs in the functional regions of the *MMP-9* gene, and found that their genotype distribution and allelic frequencies were not significantly different between patients with severe sepsis and controls or between surviving and non-surviving patients with severe sepsis [28]. We evaluated a SNP of the *MMP-16* gene because, like all MMPs, MMP-16 is a zinc-dependent enzyme and this trace element is critically important for the normal functioning of the innate and adaptive immune systems [29]. One consistent observation made in many gene expression studies is that pediatric septic shock is characterised by the widespread repression of gene families that directly participate in zinc homeostasis or directly depend on it for their normal function [30]–[34]. Moreover, the rs2664349 SNP not only seems to influence the pulmonary expression and function of MMP-16 and the risk of bronchopulmonary dysplasia in premature infants, but also the activation of MMP-2 [35], an MMP that plays a central role in monocyte chemoattraction and, consequently, in the response to infectious agents.

Among the studied SNPs in the *BPI* gene, a gene that codifies for a factor that plays an important antibacterial and antinflammatory role [36], only BPI.rs4358188-AG was associated with a reduced susceptibility to sepsis, whereas BPI rs1341023, rs5743507 and rs2232578 SNPs were apparently not important at this regard. However, other studies have led to different results. Abu-Maziad *et al.* [8] investigated three of the four SNPs evaluated in this study and found that they had no effect, whereas Michalek *et al.* [37] reported a negative association between BPI SNPs and sepsis in children aged 0–18 years in so far as GG genotype (rs4358188) of *BPI* and AG genotype (rs 5743507) were associated with increased susceptibility to severe sepsis and a negative outcome. Once again, differences in the characteristics of the patients and the ethnicity of the study population could explain the different findings.

On the contrary, the data regarding DEFβ1, an antimicrobial peptide involved in the resistance of epithelial surfaces to microbial colonisation and the regulation of the release of pro-inflammatory cytokines and adhesion molecules [38], are quite similar to the adult data reported by Chen *et al.* [39]. They studied two of the DEFβ1 SNPs evaluated in this study (rs11362 and rs17999469) and found that, as in this study, they, together with rs1800972, were associated with a reduced risk of susceptibility to sepsis and a reduced risk of severe sepsis, whereas other SNPs were closely related to an increased risk of disease and its negative evolution. These findings provide further evidence that DEFβ1 is involved in an immune response that is crucial for the pathophysiology of severe sepsis.

We found that the severity of sepsis was mainly associated with CD14 rs2569190-GG and to IL8 rs4073-AT. CD14 is a component of the lipopolysaccharide receptor molecule and serves as a central pattern recognition molecule in innate immunity. Bound to TLR4, it can activate the NF-kB signalling pathway and initiate an inflammatory response [40]. Our findings are in line with the results of a recent meta-analysis in which, after evaluating all of the available data regarding possible associations between CD14 SNPs and sepsis, it was concluded that CD14 rs2569190 is not a marker of susceptibility but is more frequent among patients with severe disease and a poor outcome, and can therefore be considered a marker of potentially severe sepsis [41].

In addition to CD14 rs2569190-GG, one SNP of the *IL8* gene was also associated with severe sepsis. This is the first demonstration that an IL8 genetic variation may condition the severity of sepsis, and conflicts with the finding of Azu-Maziad *et al.* [8] that were negative at this regard. However, it is not surprising because IL8 is a member of the chemokine family that initiates and amplifies the inflammatory processes that occur in response to a wide variety of infecting pathogen, and it has been shown that SNP rs4073-AT of the IL8 gene is associated with increased IL8 production in whole blood stimulated with lipopolysaccharides [42] and also with severe respiratory infections [43].

Finally, LTA SNPs were associated with an increased risk of sepsis due to Gram-negative rods. LTA is a mediator of the sepsis cascade, and it has been previously shown that LTA.rs1800629-AG genotype is associated with susceptibility to sepsis [44]. Although we did not find this kind of association, the greater frequency of this SNP in premature neonates with sepsis due to Gram-negative roads seems to indicate that variations of in the *LTA* gene may play a role in conditioning the development of sepsis, at least when it is potentially caused by specific infectious agents.

The finding that homogozygotes and heterozygotes for BPI (rs1341023) seem to be at increased risk of Gram-negative sepsis is surprising because other SNPs of the same gene seem to play a protective role. However, the possibility that different variations of a single gene involved in the regulation of human defences can lead to opposite results has been widely demonstrated [39].

In conclusion, this study confirms that genetic variability seems to play a role in susceptibility to, and the severity of neonatal sepsis, as well as in the risk of sepsis due to specific pathogens. However, as frequently occurs in the case of genetic studies of the associations between SNPs and clinical phenotypes, the results often conflict with previously reported. The main limitations of such investigations are the small sample sizes, the lack of simultaneous evaluations of other possibly unknown SNPs that could influence the final results, and the characteristics of the control group. However, our findings highlight the potential role of various SNPs, whose importance needs to be confirmed by further studies that should also evaluate the consequences of mutations on gene expression. If confirmed, the new finding regarding *MMP-16* gene could significantly contribute to a better understanding of premature infants' defences against bacterial invasion and aid the development of more effective therapeutic measures. Preliminary data suggest that targeting MMPs may be beneficial in infectious disease, particularly the administration of direct inhibitors in order to regulate enzyme activity and target the signalling pathways that up-regulate MMP expression [45], [46].
